# An anticipatory mechanism enhances the cooperative behaviors of quorum sensing mutants in *Pseudomonas aeruginosa*

**DOI:** 10.1371/journal.ppat.1013046

**Published:** 2025-04-15

**Authors:** Min Yuan, Huifang Qiu, Xiaoqing Zhou, Weijun Dai

**Affiliations:** Integrative Microbiology Research Center, College of Plant Protection, South China Agricultural University, Guangzhou, China; University of Colorado Anschutz Medical Campus, UNITED STATES OF AMERICA

## Abstract

Social interactions substantially influence the dynamics and functions of microbial communities. Cooperative behaviors serve to benefit populations, yet they are often exploited by cheating cells, thus creating a conflict between individuals in the microbial population. However, the underlying mechanisms by which cooperative behaviors are stabilized are incompletely elucidated. Here, we used quorum sensing (QS) as a model of cooperation, and functionally studied QS regulator LasR variant strains in the context of cooperative behaviors. We found that a LasR228 variant strain, bearing a non-conserved substitution in LasR, exhibited minimal LasR-dependent phenotypes. However, the function of this LasR228 variant strain was restored by inactivation of the transcriptional repressor PsdR, and the phenotypes of this variant strain were similar to the parental strain. Furthermore, we illustrate a post-transcriptional regulatory mechanism responsible for the activation of the LasR228 variant. Unlike LasR228, the PsdR-null-LasR228 strain demonstrated cooperative behaviors in competition with the LasR-null strain. Since *psdR* mutations precede the emergence of LasR variants in the evolution of *P. aeruginosa* using casein broth, this PsdR-mediated cooperative mechanism serves as an anticipatory control against potential cheating LasR variant strains. Additionally, our cell-killing assay showed that the cooperative PsdR-null-LasR228 strain was associated with increased bacterial pathogenicity to eukaryotic host cells. In conclusion, our study reveals the functional plasticity of LasR variants, which can be modulated by secondary mutations, affecting cooperation and conflict within populations. Our identification of a novel cooperative molecular mechanism offers insight into the maintenance of cooperation within microbial communities.

## Summary

This research uncovers a new mechanism that stabilizes cooperation in microbial populations by studying quorum sensing (QS) in *Pseudomonas aeruginosa*. Our findings identified a regulatory pathway where the PsdR protein modulates the function of a QS regulator variant (LasR228), influencing cooperative behaviors and pathogenicity. By inactivating PsdR, the variant’s cooperative behaviors were enhanced, facilitating the production of public goods. This finding highlights how microbial communities can adapt through secondary mutations to preserve cooperative dynamics, which are essential for their survival and pathogenic traits. Understanding these mechanisms provides valuable insights into microbial evolution and the potential development of therapies targeting microbial cooperation to mitigate infections.

## Introduction

Microbial populations can exist as a complex community wherein cells communicate, compete, and cooperate with each other [1]. Social interactions between cooperative cells and exploitative individuals play important roles in shaping the dynamics and functions of microbial populations [2]. These interactions can determine the community composition and various physiological processes [3,4], including pathogenesis [5,6], antibiotic resistance [7] and host infection [8,9]. Understanding the molecular network of social interactions is thus fundamental to the study of evolutionary biology.

However, cooperative interactions introduce conflicts within microbial communities, because cooperative behaviors benefit the entire population at the expense of individual cells [10,11]. Indeed, the coexistence of cooperators and cheaters within microbial populations is rather common [12], suggesting the existence of mechanisms to resolve or deter these conflicts. Studies have identified molecular mechanisms that safeguard cooperative behavior from exploitation [13]. These mechanisms include the partial privatization of public goods accessible only to producers [14,15], the policing of cheaters through the production of self-immune or resistant toxic chemicals [16,17], the facultative cooperation through selectively producing and utilizing public goods [18], and the co-regulation of private and public goods [19]. Kin recognition serves as another mechanism governing social behavior by discriminating relatedness [20]. These diverse cooperative mechanisms confer robustness to cooperation and stabilize populations under specific environmental constraints.

*Pseudomonas aeruginosa*, an opportunistic human pathogen, is well known for causing severe infections in immunocompromised patients [21]. *P. aeruginosa* uses a cell-cell communication system called quorum sensing (QS) to regulate population behaviors, including production of virulence factors [22]. QS in *P. aeruginosa* involves two systems, LasI-R and RhlI-R, that use acyl-homoserine lactone (AHL) signals, and a third system, the Pseudomonas quinolone signal (PQS) system [23,24]. The AHL systems involve production of the signals N-3-oxo-dodecanoyl homoserine lactone (3OC_12_-HSL) and N-butanoyl homoserine lactone (C_4_-HSL) by the signal synthases LasI and RhlI, respectively. These signals bind to the transcription factors LasR and RhlR, and the signal-bound transcription factors activate the transcription of dozens of genes. Within *P. aeruginosa* populations, social interactions significantly influence various bacterial functions [25]. Notably, when *P. aeruginosa* grown in media containing casein as the sole carbon and energy source, the emergence of QS mutants is a common phenomenon [26,27]. These mutants harbor variants in the gene encoding LasR and are deficient in producing elastase, a protease that is a public good. Consequently, they act as cheaters, exploiting the QS-dependent extracellular elastase produced by cooperative cells, which is essential for breaking down casein to provide carbon and energy for bacterial growth [26,27]. This cheating strategy confers a relative growth advantage to the mutants, with their prevalence sometimes reaching 25% within evolving populations [28]. The LasR mutants impose a metabolic burden on the population, resulting in reduced cell density and public good yields for the entire community [26]. In theory, this intrusion of cheating LasR mutants may eventually cause a so-called “tragedy of the commons” that leads to the collapse of a whole population. However, instances of population crashes due to the emergence of LasR cheater mutants are rare [28]. This population equilibrium implicates hitherto unknown cooperative mechanisms that constrain LasR mutants and contribute to population stabilization.

Besides *lasR* mutations, mutations in the *psdR* gene also commonly appear when *P. aeruginosa* is cultivated in casein broth [28,29]. The *psdR* gene encodes an XRE-cupin family protein that contains an N-terminal helix-turn-helix xenobiotic response element (XRE) domain and a C-terminal cupin sensor domain [30]. Similar to other characterized members of the XRE-cupin transcriptional regulators, PsdR was described as a local transcriptional regulator, suppressing the transcription of its neighboring genes, *mdpA* and *dppA3* [30]. These two genes encode a metallo-dipeptidase [31] and a small peptide transporter [32], respectively. By downregulating the expression of *mdpA* and *dppA*3, PsdR effectively controls the transport and processing of dipeptides in *P. aeruginosa* [28,30,31]. Notably, *psdR* mutations arise quickly and become prevalent within the population, preceding the appearance of the LasR mutants. As described by previous findings, these PsdR mutants confer a fitness advantage termed “non-social” within the population [28]. The prevalence of PsdR variants is attributed to their enhancement of intracellular dipeptide utilization, conferring a private benefit only to cells carrying this mutation [28].

We recently uncovered that PsdR is also a negative regulator of QS [33]. In addition to the *mdpA* and *dppA3* genes, PsdR directly binds to the *lasR* promoter and represses its transcription. Furthermore, PsdR did not bind to the same promoter when the PsdR-binding site was deleted. This direct regulation of *lasR* underscores PsdR’s role in modulating the QS circuit primarily through the Las QS system. This finding led us to infer that PsdR may potentially influence QS-related cooperative behavior, other than the previously reported non-social benefit. In the present study, we identified various LasR variants through *P. aeruginosa* in vitro evolution. We investigated the impacts of PsdR inactivation on the functions and social dynamics of a *P. aeruginosa* PAO1 strain containing a LasR variant (A228V, named LasR228). Our findings revealed that PsdR inactivation substantially stimulated LasR-dependent phenotypes in this variant strain, resulting in QS activation and QS-controlled products. Furthermore, we elucidated the activation mechanism of the LasR228 variant, revealing that the increased expression of the *lasR* variant induced by inactive PsdR triggers the Las QS positive feedback loop. The QS activation in the PsdR-LasR228 variant strain resulted in re-acquisition of cooperative traits. Moreover, this variant strain also demonstrated enhanced cell cytotoxicity effects on host mammalian cells. Finally, we showed that this PsdR-mediated QS activation and promotion of cooperative behaviors occurred with other LasR variant strains isolated from natural environments.

## Results

### Identification and characterization of LasR variants in *P. aeruginosa* evolution

To characterize LasR variants that emerge during the evolution of *P. aeruginosa*, we analyzed LasR variant data from previous publications [26,34,35]. In these papers, LasR variant isolates were commonly obtained from in vitro evolution experiments using the laboratory *P. aeruginosa* wild-type strain PAO1. PAO1 was passaged and evolved in minimal medium with casein as the sole carbon and energy source (“casein broth”) [28,29]. These LasR variant isolates harbor diverse mutations in the *lasR* gene, including deletions and single nucleotide substitutions, resulting in either protein truncations or single amino acid changes (S1 Table). Truncations are very likely to give rise to an inactive LasR protein, while single amino acid changes may have variable effects on LasR functionality [36]. To further investigate the impacts of single amino acid substitutions on LasR functionality, we conducted a sequence alignment using LasR protein homologs from different bacterial species (S2 Table). We found that changed residues in these LasR variants are distributed across the ligand-binding domain (LBD) and DNA-binding domain (DBD) of the LasR protein (S1 and S2 Figs). Moreover, conservation analysis of residues showed that single amino acid substitutions in these LasR variants are either conserved or non-conserved (S3 Fig). This diverse profile of positional substitutions implicates the varied functionalities of these LasR variants. Given that these LasR variant mutants were selected in the course of *P. aeruginosa* QS evolution [26,34,35], we predicted that they might carry out diverse functional roles in social interactions within the evolving bacterial community.

### Inactivating PsdR enhances the proteolytic phenotype of the LasR228 variant strain

We noticed that a particular LasR variant (A228V, designated as LasR228) strain, consistently maintained 10 ~ 30% of the *P. aeruginosa* population during the evolutionary process in multiple instances [26,28]. This prevalence hints at its competitive advantage within the evolving population. Intriguingly, unlike the LasR-null mutant, this variant strain was reported to exhibit a near-wild-type level of LasR-dependent activity due to its active LasR228 variant protein [26]. To functionally characterize this LasR variant, we introduced corresponding single nucleotide substitution into the strain PAO1, generating the PAO1 LasR228 variant strain. Given the common occurrence of *psdR* mutations during *P. aeruginosa* evolution in a casein broth [28], we also deleted the *psdR* gene in the LasR228 variant strain, resulting in PsdR-LasR228. In addition, we engineered a PAO1 LasR variant strain containing a substitution at a highly conserved site, designated as LasR73 (S3 Fig). This variant strain serves as a LasR-null-like negative control.

**Fig 1:**
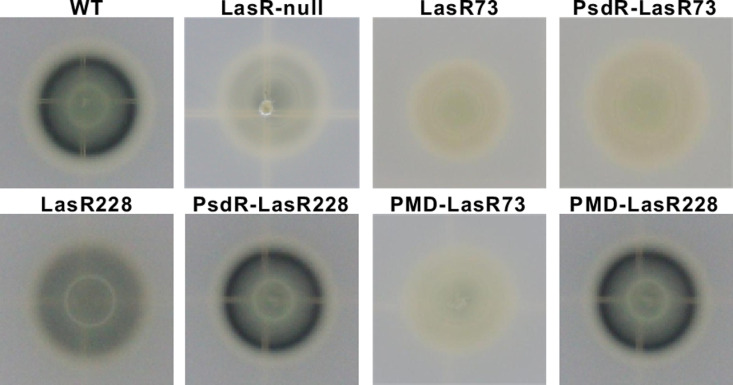
The proteolytic activity of constructed PAO1 LasR variant strains. Equal numbers of bacteria of the indicated strains were spotted on skim milk plates to estimate proteolytic activity resulting in transparent proteolytic zones (halos). Pictures were taken 24 h later. WT, wild-type PAO1 strain; LasR-null, LasR-null mutant; LasR73, LasRD73E mutant; PsdR-LasR73, PsdR-LasRD73E mutant; LasR228, LasRA228V mutant; PsdR-LasR228, PsdR-LasRA228V mutant; PMD-LasR73, PsdR-MdpA-DppA3-LasRE73E mutant; PMD-LasR228, PsdR-MpdA-DppA3-LasRA228V mutant.

We next evaluated the LasR-dependent extracellular proteolytic activities of these variant strains. First, we observed a similar growth in LB across these variant strains (S4 Fig). In our strains, we found that only the PsdR-LasR228 strain displayed a near-wild-type level of proteolytic activity, as reflected by the pronounced halo zones on the skim milk agar plate (Fig 1). In contrast, the LasR228 strain with an intact *psdR* exhibited a rather faint halo zone (Fig 1). This result indicates that LasR activity is substantially stimulated in the PsdR-LasR228 strain but down-regulated in the LasR228 strain. Notably, this heightened LasR activity in the PsdR-LasR228 strain occurs independent of the PsdR-mediated repression of dipeptide uptake, as deletion of the *mdpA* and *dppA3* genes did not affect proteolytic activity in either the PMD-LasR228 or PMD-LasR73 strains (Fig 1). As expected, both LasR73 and PsdR-LasR73 strains displayed LasR-null-like proteolytic activity (Fig 1), indicating the functional inactivity of LasR brought by the D73E substitution. Taken together, our findings reveal that with or without a functional PsdR, the LasR228 variant strain exhibits distinct LasR-dependent phenotypes. The absence of PsdR leads to an active LasR228 variant in the PsdR-LasR228 strain, while this variant was functionally detrimental in the LasR228 variant strain where a functional PsdR is present.

### QS activity in the PsdR-LasR228 strain

**Fig 2:**
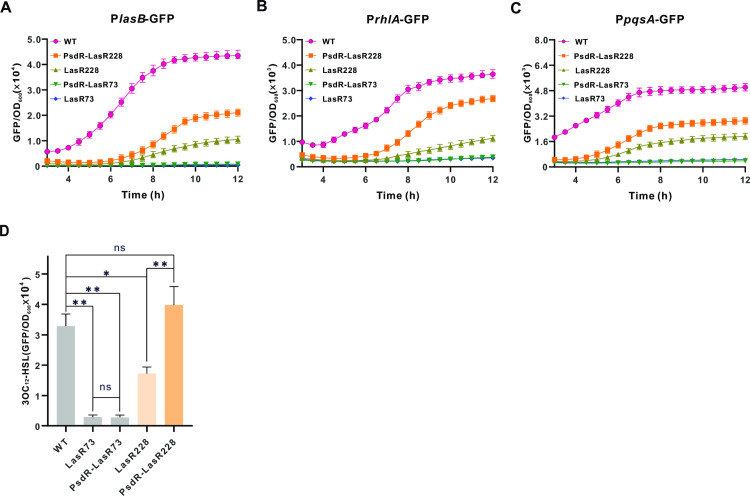
QS-related activities in shown strains. **(A-C)** Las-(A), Rhl- (B) and PQS-responsive **(B)** QS activities of the shown strains. Las-, Rhl- and PQS-responsive QS activities are reflected by the fluorescence levels of the expressed reporters P*lasB*-GFP, P*rhl*Α-GFP and P*pqsA*-GFP, respectively. Fluorescence values, expressed as relative fluorescence units (GFP/OD_600_), were obtained from bacteria cultured for 18 h. **(D)** the relative concentrations of 3OC_12_-HSL in the shown strains. Data are presented as means ± SD (*n* = 3, *t*-test). *, *P* < 0.05; **P < 0.01; ***, *P* < 0.001 (t-test).

Considering LasR’s role as a master regulator in the QS hierarchy controlling Las, Rhl and PQS systems [22], it is likely that the activated LasR228 variant further amplifies QS circuit activation in the PsdR-LasR228 strain. To address this question, we examined QS-regulated gene expression. We used the promoter-GFP reporter system (P*lasB*-GFP, P*rhlA*-GFP and P*pqsA*-GFP) to evaluate transcription levels of representative genes regulated by Las, Rhl and PQS QS systems. Consistent with the proteolytic phenotype (Fig 1), the PsdR-LasR228 strain carrying these QS reporters displayed significantly heightened fluorescence compared to the QS reporter-containing LasR228 strain bearing a functional PsdR, indicating a robust QS activation in the PsdR-LasR228 strain (Fig 2A–2C). By contrast, both LasR73 and PsdR-LasR73 strains exhibited similarly low levels of fluorescence, indicating inactive QS circuits in these strains. Accordingly, the PsdR-LasR228 strain produced greater concentrations of the QS signal 3OC_12_-HSL than the LasR228 strain as well as the LasR73 and PsdR-LasR73 strains (Fig 2D). In conclusion, deletion of *psdR* induces pronounced QS activation in the PsdR-LasR228 strain but not in the LasR-null-like PsdR-LasR73 strain.

### Increased transcriptional expression results in the functional activation of the LasR228 variant

**Fig 3:**
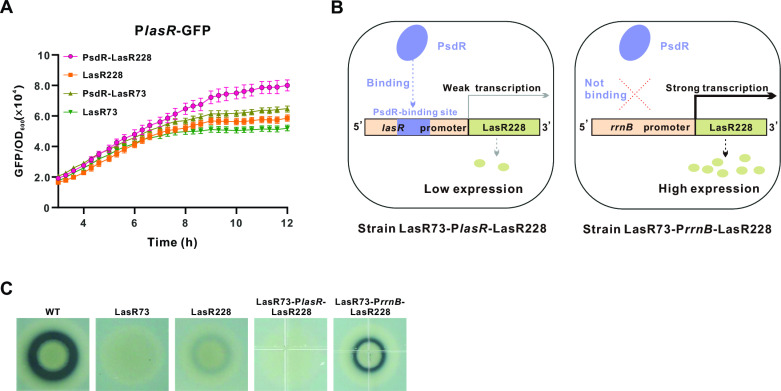
Increased transcriptional expression in the LasR228 variant strain leads to functional activation. **(A)** *lasR* transcription in indicated strains. The transcriptional level of *lasR* is estimated by the fluorescence signal of the P*lasR*-GFP. Fluorescence values, expressed as relative fluorescence units (GFP/OD_600_), were obtained from bacteria cultured for 18 h. **(B)** Illustration of the LasR228 variant constructs bearing distinct promoter regions and the resulting strains. **(C)** Proteolytic activity of strains using skim milk agar plates. LasR73-P*lasR*-LasR228, the LasR73 strain containing the miniTn7-PlasR-LasR228 construct; LasR73-P*rrnB*-LasR228, the LasR73 strain containing the miniTn7-P*rrnB*-LasR228 construct.

We next explored the molecular mechanisms that lead to the functional activation of the LasR228 variant in the PsdR-LasR228 strain. We recently uncovered that in addition to the local repression of its two neighboring *mdpA* and *dppA3* genes [30,31], PsdR can inhibit the transcription of *lasR* by directly binding to its promoter [33]. We therefore inferred that *lasR* derepression by PsdR inactivation may lead to the activation of the LasR228 variant. We used the reporter construct (P*lasR*-GFP), in which the promoter of *lasR* was fused to GFP, to estimate the level of *lasR* transcription. Consistent with our hypothesis, there was a significant increase in fluorescence, reflecting robust *lasR* expression, in the PsdR-LasR228 strain (Fig 3A). By contrast, other assayed strains showed comparatively low fluorescence levels. These findings demonstrate a positive correlation between *lasR* expression and LasR228 variant functionality.

To further elucidate the role of transcriptional modulation in the functional activation of LasR228 variant, we assessed the impacts of different promoter sequences on the activity of the LasR228 variant. We generated two *lasR* variant constructs: miniTn7-P*lasR*-LasR228 and miniTn7-P*rrnB*-LasR228. In the miniTn7-P*lasR*-LasR228 construct, the *lasR* variant retains its native promoter that harbors an identified PsdR-binding site. In the other construct, this promoter was replaced by P*rrnB*, a bacterial ribosomal promoter [37], to which PsdR does not bind (Fig 3B). These constructs were mobilized into the LasR73 strain, resulting in strain LasR73-P*lasR*-LasR228 and strain LasR73-P*rrnB*-LasR228. The activities of the LasR228 variants were then visualized by protease-catalyzed zones on skim milk agar plates. Similar to LasR-null, no visible protease-catalyzed zones could be observed in the LasR73-P*lasR*-LasR228 strain (Fig 3C), indicative of a non-functional LasR228 variant under the condition of PsdR repression. This outcome is expected because the expression of the LasR228 variant is repressed by PsdR. Intriguingly, the LasR73-P*rrnB*-LasR228 strain exhibited visible protease-catalyzed zones, suggesting functional activation of the LasR228 variant facilitated by the promoter P*rrnB* (i.e., without PsdR repression). In summary, our results provide evidence that elevated transcriptional expression results in the functional activation of the LasR228 variant.

### The role of the Las positive feedback loop in the functional activation of the LasR228 variant

**Fig 4:**
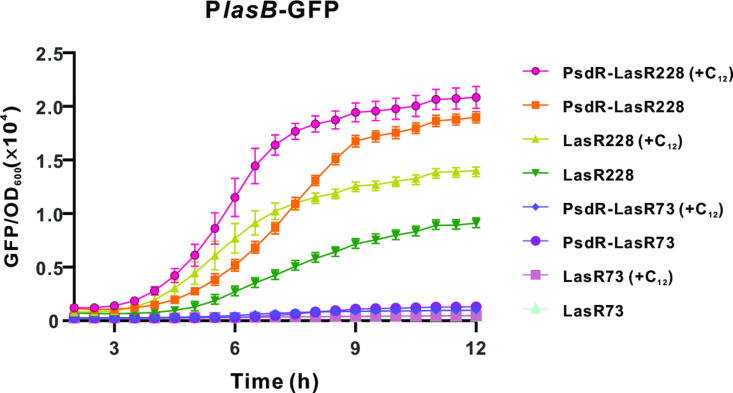
Influences of 3OC_12_-HSL on the LasR-responsive activity of LasR variant strains. The LasR-responsive activity of the indicated strains. LasR-responsive activity is reflected by the fluorescence levels of the expressed reporter P*lasB-*GFP. Fluorescence values, expressed as relative fluorescence units (GFP/OD_600_), were obtained from bacteria cultured with or without supplementation of 3OC_12_-HSL for 18 h. Data are presented as means ± SD (*n* = 3, *t*-test).

In the Las QS system, signal-bound LasR activates the transcription of *lasI*, which encodes the signal synthase. This means that the expression of *lasR* and/or *lasI* genes stimulates their own expression, forming a positive Las feedback loop [38,39]. Based on our findings above, we inferred that unlike the LasR73 variant, the LasR228 variant is not completely inactive and retains a basal level of functionality. The enhanced transcription of the *lasR* variant may thus trigger the Las QS positive feedback loop, consequently leading to the functional activation of the LasR228 variant. Therefore, alternative mechanisms promoting the Las feedback loop could potentially result in the functional activation of the LasR228 variant. To test this idea, we examined the effects of the Las QS signal 3OC_12_-HSL on the LasR228 variant. Exogenous 3OC_12_-HSL was supplied to LasR variant derivative strains, and their activities were measured using the P*lasB*-GFP reporter. Consistent with our hypothesis, adding 3OC_12_-HSL increased LasR-dependent gene transcription in the LasR228 variant derivative strains (Fig 4). In contrast, LasR73 strains, regardless of the presence of a functional PsdR, did not respond to the addition of 3OC_12_-HSL. These findings underscore that through the facilitation of the Las positive feedback loop, the LasR228 variant can be functionally activated.

### Cooperative roles of the PsdR-LasR228 strain during *P. aeruginosa* evolution

**Fig 5:**
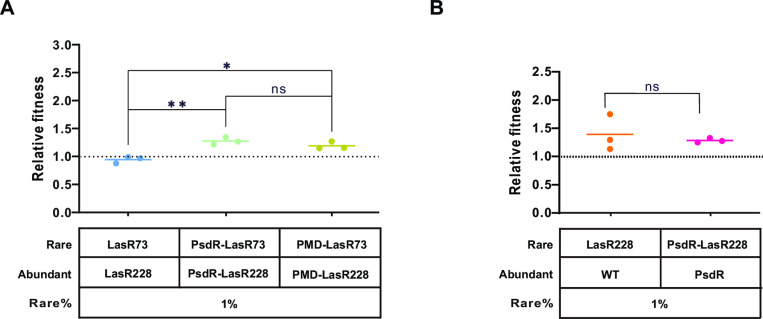
The relative growth fitness of LasR variant strains. **(A-B)** The relative growth fitness of indicated strains. Each Gm-labeling strain was co-cultured with the designated strain at a start ratio of 1:99 and grown in casein broth for 24 h. Colonies were enumerated and relative fitness was computed as the ratio of Malthusian growth parameters (*w*). A one-way ANOVA with Bonferroni posttest or *t*-test was used for statistical analysis. *, *P* < 0.05; **, *P* < 0.01. ns, not significant.

Given that QS promotes cooperative behaviors [40], we investigated whether QS activation in the PsdR-LasR228 strain influences its social interactions. To address this question, we first conducted monoculture experiments in casein broth. Both the wild-type strain and PsdR-LasR228 strain exhibited robust growth, whereas the LasR228 strain displayed limited growth. In contrast, the LasR73 strain, regardless of PsdR functionality, could not grow independently in casein broth (S5 Fig). Next, we assessed the competitive fitness of LasR228 and LasR73 strains, with or without a functional PsdR, when cocultured in casein broth. The competition experiments revealed no significant fitness advantage for either functional PsdR-containing strain (LasR228 or LasR73) over its counterpart (Fig 5). However, the PsdR-LasR228 strain was outcompeted by the PsdR-LasR73 strain, suggesting that the PsdR-LasR228 strain plays a restricted cooperative role specifically when co-cultured with the LasR-null-like variant. In this scenario, the QS-inactive PsdR-LasR73 strain, which can’t grow independently in casein broth, had a fitness advantage by exploiting the public goods (such as extracellular proteases) produced by the QS-activated PsdR-LasR228 strain. Importantly, this cooperative role of the PsdR-LasR228 strain is independent of the PsdR-mediated dipeptide regulation (Fig 5A). On the other hand, both LasR228 and PsdR-LasR228 strains still exhibited cheating behaviors in competition with their respective parental strains (Fig 5B). This suggests that *psdR* mutations increase the fitness tolerance of the LasR228 strain relative to the LasR73 strain, although not to the same extent as observed in the wild-type strain. Taken together, our competition data, in combination with the protease production results (Figs 1 and 2), support the idea that the LasR73 strain can act as a cheater by exploiting proteases produced by the LasR228 strain during co-culture. Furthermore, the LasR228 strain can exploit proteases secreted by the wild-type strain. In this sense, the LasR228 strain may be either cooperative or exploitative depending on the context.

### Cooperative PsdR-LasR228 strain increases bacterial pathogenesis

**Fig 6:**
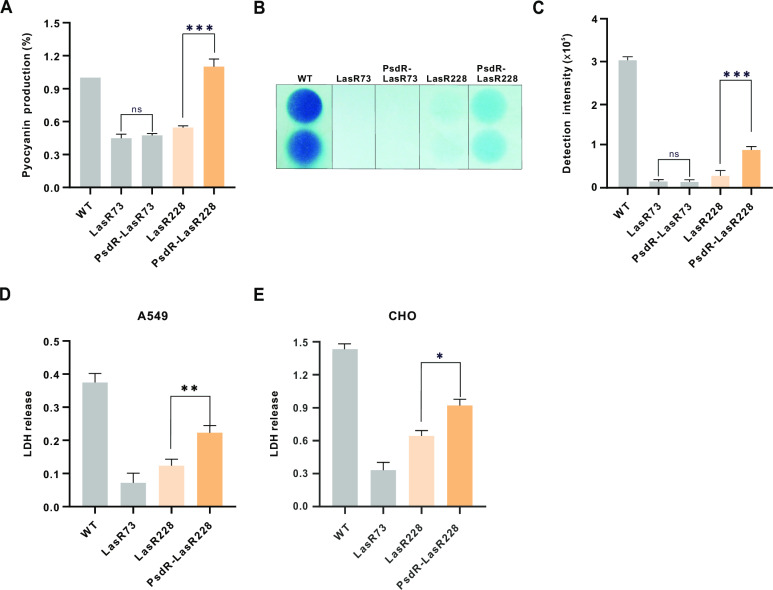
Increased bacterial pathogenesis in the PsdR-LasR228 variant strain. **(A)** Pyocyanin production in designated strains. Pyocyanin production (OD_695_/OD_600_ values) was quantified for each strain. **(B)** Cyanide levels of indicated colonies. Cyanide-sensitive filter papers were photographed after post bacterial inoculation 18-24 h in the 24-well plate at 37 °C. **(C)** Quantification of cyanide production in (B) using ImageJ software. **(D-E)** The killing assay with human lung carcinoma A549 (D) and CHO (E) cells was performed with equal amounts of indicated strains. After incubation for 6 h, the release of cytosolic lactate dehydrogenase (LDH) from infected cells was detected. The released amount of LDH inoculated with the wild-type strain was set to 100%. Data represent means ± SD (*n* = 3). A one-way ANOVA with Bonferroni posttest was used for statistical analysis. *, *P* < 0.05; **, *P* < 0.01.

We next asked the question of the impacts of the cooperative PsdR-LasR228 strain on bacterial pathogenesis. We first evaluated the QS-dependent virulence products in the PsdR-LasR228 strain. As shown in Fig 6A–6C, the PsdR-LasR228 strain showed enhanced levels of QS-controlled virulence factors, pyocyanin and hydrogen cyanide, compared to the LasR228 strain. To further assess the effects of the PsdR-LasR228 strain on bacterial virulence, we measured the ability of these variant strains to kill Chinese hamster ovary (CHO) cells and human lung adenocarcinoma A549 cells. Cell death was quantified by monitoring the release of cytosolic lactate dehydrogenase (LDH) into the cytosol. Consistent with its enhanced production of QS-dependent virulence factors, the PsdR-LasR228 strain displayed significantly increased cell cytotoxicity compared to its parental LasR228 strain, reaching about half of the level of the wild-type strain (Fig 6D and 6E). Overall, our findings demonstrate that the cooperative PsdR-LasR228 strain not only contributes to population stabilization but also increases bacterial pathogenicity compared to the LasR228 strain.

### LasR228-like variant strains are common in native environments

To explore whether LasR228-like variant strains are common in native environments, we sought to identify LasR variants isolated from diverse sources. Using the PAO1 LasR as a query sequence, 6697 LasR sequences of *P. aeruginosa* strains from clinical and environmental sources were retrieved from the Pseudomonas Database [41]. Among these identified sequences, 40.9% of the LasR sequences contain changes including insertions, deletions and single amino acid substitutions. Insertions and deletions of amino acids are most likely to result in a completely inactive LasR protein due to frameshift. Therefore, we only focused on those LasR variants that contain only single amino acid substitution (S3 Table). We noticed that the LasR228 variant has one hit (*Pseudomonas aeruginosa* AZPAE15027) among these analyzed strains.

We selected a LasR228-like variant (E196D, named LasR196) for the following investigation. Similar to LasR228, this LasR196 variant, which has 10 hits among analyzed strains (S3 Table), contains a substitution at a non-conserved site within the DBD. This *lasR* variant was introduced into the PAO1 genome, generating the PAO1 LasR196 variant strain. Similar to the PsdR-LasR228 strain, PsdR inactivation in the LasR196 strain stimulated LasR-dependent LasB activity as well as QS circuit activation, as determined by a series of reporter assays (S6 Fig). This indicates functional activation of the LasR196 variant. Moreover, the activation process of the LasR196 variant closely resembles that of the LasR228 variant, both involving a similar post-transcriptional mechanism. Either upregulated *lasR* transcription via PsdR inactivation or supplementation of 3OC_12_-HSL markedly elevated LasR-dependent activities in the LasR196 strain (S7 and S8 Figs). Consequently, the QS-activated PsdR-LasR196 strain, but not the QS-detrimental LasR196 strain, was outcompeted by the LasR73 strain in the competition growth experiment (S9 Fig). This indicates that the PsdR-LasR196 strain may exhibit a relatively cooperative behavior when specifically competing with the LasR-null-like LasR73 strain. These findings suggest that LasR228-like variants are common among strains obtained from native environments. The functional plasticity of these LasR variants allows functional activation by secondary adaptive mutations, leading to QS activation and corresponding cooperation promotion in their native environments.

## Discussion

**Fig 7:**
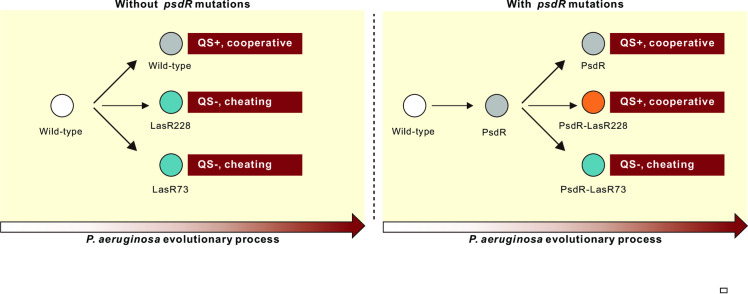
Schematic representation of the evolutionary trajectory during *P. aeruginosa* evolution. Impacts of *psdR* mutations on the social roles of LasR variant mutants during *P. aeruginosa* evolution in the casein broth. QS-, QS inactive; QS+, QS-active.

In the present study, we uncover a novel regulatory mechanism (Fig 7) that facilitates cooperation in microbial populations. We discovered that the inactivation of PsdR leads to the transcriptional upregulation of the QS master regulator gene *lasR*, thereby resulting in the functional activation of some QS-detrimental LasR variants. This activation enables these LasR variants to produce public goods, functioning as cooperators relative to LasR-null mutants. Our work highlights how this newly identified cooperative mechanism directly converts some potential LasR variant cheaters into cooperators under specific conditions. Several cooperative mechanisms have been described to constrain the emergence of cheaters, such as public goods privatization [14,15], policing [16,17] and facultative cooperation [18]. These mechanisms work by diminishing the fitness advantage of existing cheaters while enhancing that of cooperators. The mechanism that we describe here, involving PsdR, is distinct from these known cooperative mechanisms. The *psdR* mutations precede the emergence of *lasR* variants in the course of *P. aeruginosa* QS evolution, and quickly spread through the whole population [28]. Therefore, each *lasR* variant is inherently pre-equipped with *psdR* mutations. Thus, the PsdR-mediated cooperative mechanism can be viewed as a strategy employed by the bacterium for promoting cooperation within the population under certain environments. In summary, our detailed dissection of this new cooperative mechanism at the molecular level significantly advances our understanding of social interactions within microbial communities. Our findings provide a new perspective regarding how bacteria can resolve the conflict of maintaining cooperative behaviors that are vulnerable to exploitation by cheating.

In general, the functional characterization of LasR variants involves introducing specific *lasR* mutations

into laboratory strains, such as PAO1. These mutant strains are then phenotypically assessed in comparison with LasR-null strains under controlled laboratory conditions. This standard approach allows for the separation of LasR variants from their native environments, facilitating the understanding of how specific mutations impact LasR functions. Employing this method, previous studies have shown a preference for non-synonymous substitutions in isolated LasR variants [9]. However, our current investigations revealed that with or without *psdR* mutations, different LasR variants exhibit distinct LasR activity and corresponding LasR-dependent phenotypes (e.g., LasR73 vs PsdR-LasR73 and LasR228 vs PsdR-LasR228). In other words, the activities of LasR variants can be modulated by other adaptive mutations to varying degrees (in this case, *psdR* mutations). Therefore, the actual physiological functions of LasR variants and their resulting social roles within a population may be masked and challenging to characterize if only specific *lasR* mutations are considered without accounting for adaptive mutations in *psdR* or other genes. Asfahl et al. reported a PAO1 isolate named HC, containing mutations in *psdR*, *lasR* (encoding the same LasR228 variant) and PA2408, from *P. aeruginosa* evolution in casein broth [28]. This HC isolate exhibited partial QS-positive phenotypes, which were primarily defined by the LasR variant itself. In contrast, our work illustrates that the activity of the LasR228 variant is relatively subdued compared to the wild-type PAO1 strain, and it was the *psdR* mutation that fully restored the activity of the LasR228 variant and the corresponding QS-related phenotypes. Based on our findings, we suggest that uncovering the activity of transcription factor variants requires taking into account the roles of related adaptive mutations within specific environments.

Our study demonstrates that the LasR228 variant can be activated by acquiring adaptive mutations in the *psdR* gene. The inactivation of PsdR derepresses *lasR* transcription, thereby increasing the abundance of *lasR* variant transcripts. Since the LasR228 variant is not completely inactive and maintains a baseline level of activity, the elevated expression of the LasR variant may surpass the threshold required for Las QS activation, thus triggering the well-known Las positive feedback loop [38,39]. This ensuing feedback loop ultimately leads to the functional activation of the LasR228 variant. In support of this proposed model, our current study demonstrated that supplementation with the LasR signal 3OC_12_-HSL also resulted in the functional activation of the LasR228 variant. In summary, our findings unveil the functional plasticity of a LasR variant, which can be modulated through a post-transcriptional regulatory mechanism. Intriguingly, similar incompletely inactive LasR variants were also identified in *P. aeruginosa* isolates obtained from cystic fibrosis (CF) patients with chronic infections [36], suggesting that similar variants are rather common in some native conditions. Given that Las QS regulates the expression of approximately 10% of genes encoded in the genome and controls various bacterial cellular physiological processes and virulence phenotypes [42], this post-transcriptional regulatory mechanism may facilitate a functional switch for these LasR228-like variants, enabling rapid bacterial adaptations to changing environments.

As a member of the XRE-cupin family, PsdR was previously characterized as a local transcriptional repressor [28,30,31]. Its inactivation results in the derepression of neighboring *mdpA* and *dppA3* genes, thereby conferring absolute growth fitness to the bacterium without affecting the expression of public goods [28]. Consequently, through its regulation of dipeptide uptake and transport [28], adaptations in *psdR* were initially understood to have a primarily non-social function in *P. aeruginosa* evolution. However, beyond its local regulator role [28,30,31], our recent work extends the understanding of the biological functions of PsdR as a potential global transcriptional regulator, uncovering that it is indeed a QS negative regulator that directly represses *lasR* expression [33]. In this study, we further demonstrate that the inactivation of PsdR stimulated the activity of various LasR variants, resulting in the activation of QS circuits as well as heightened QS-controlled public goods. This results in increased cooperation among LasR variant strains in environments where both LasR variant strains and LasR-null mutants coexist. In conclusion, our current research reveals a cooperative role of *psdR* adaptations in *P. aeruginosa* evolution. The *psdR* adaptations not only have a non-social function as described previously [28], but also carry out a social role contributing to the cooperative growth during the *P. aeruginosa* evolutionary process. Our findings provide insight into the rapid sweep of *psdR* adaptations throughout the *P. aeruginosa* population during evolution.

Our research underscores an important role of PsdR in shaping social interactions within *P. aeruginosa* communities. Inactivation of PsdR enhances cooperative behaviors among LasR variants. Conversely, upregulating PsdR expression may facilitate cheating behaviors among LasR variants, leading to reduced population stability and potential collapse. Therefore, regulating PsdR expression or activity may offer a new avenue to modulate social interactions within *P. aeruginosa* populations. PsdR is a typical member of the XRE-cupin family, and this provides a promising avenue for PsdR manipulation. In the XRE-cupin family, the XRE domain binds to the promoter DNA to regulate target gene expression, while the cupin domain senses small signal molecules [43]. Each cupin domain forms a unique signal-sensing pocket, conferring substrate specificity to interactions [30]. For example, proteins in the XRE-cupin family, such as PauR from *P. aeruginosa* and PauR from *E. coli*, respond selectively to diamines putrescine and putrescine, respectively [44,45]. Leveraging this substrate specificity, PsdR-targeted small molecules could be employed to artificially interfere with PsdR binding to target genes, thereby modulating PsdR-mediated social interactions. Ultimately, through the modulation of PsdR with specific small molecules, our findings point to a novel therapeutic avenue for combating pathogenic infections by targeting social interactions within pathogen populations.

## Materials and methods

### Bacterial strains and growth

*P. aeruginosa* strain PAO1 was grown at 37 °C in Lysogeny broth (LB) containing 10 mg/mL tryptone, 5 mg/mL yeast extract and 10 mg/mL NaCl. LB was buffered with 50mM 3-(N-morpholino) propanesulfonic acid, pH 7.0 (LB-MOPS broth). In some experiments, *P. aeruginosa* was grown on M9 medium [26] supplemented with 1% sodium caseinate (C8654, Sigma-Aldrich, New Zealand) (casein broth) or 0.5% casamino acids (A100851, Sangon Biotech, Shanghai, China) (CAA medium) as the sole source of carbon and energy. *Escherichia coli* was grown in LB at 37 °C. Unless otherwise specified, bacteria were grown in 14-mm FALCON tubes containing 3 mL of medium, with shaking (250 rpm) at 37 °C. Colonies were grown on LB agar or *Pseudomonas* Isolation agar (PIA) (1.5% agar). Bacterial strains used in this study are listed in S5 Table.

### Construction of *P. aeruginosa* mutants

Gene deletions were generated using a homologous recombination exchange approach as described previously [46]. Briefly, 1000 bp DNA flanking of the target gene were PCR-amplified and cloned into the pGEX2 vector (Gentamycin resistance, Gm) [46,47] with the Vazyme ClonExpress II One Step Cloning kit (Vazyme Biotech, Nanjing, China), generating pGEX-flanking constructs. The pGEX-flanking construct was mobilized into the *P. aeruginosa* strain by triparental mating with the *E. coli* PRK2013 strain (Kanamycin resistance, Km). Deletion mutants were initially selected on PIA containing 100 μg/mL Gm and counter-selected on LB agar containing 10% sucrose. All mutants were confirmed by PCR amplification and subsequent DNA sequencing. Primers used in this study are listed in Supplementary S4 Table.

### Reporter assay

The primers used for QS reporter constructs (P*lasB*-GFP, P*rhlA*-GFP, P*pqsA*-GFP, P*lasR*-GFP) are listed in S4 Table. QS reporter plasmids were transferred into PAO1 and derivatives either by electroporation or mating, and selected on the PIA plate (Gm50). PAO1 strains bearing QS reporter plasmids were grown in LB-MOPS broth (Gm50) for 12 h, then diluted to CAA medium (OD_600_ ≈ 0.02) and subsequently transferred to 96-well plates (200 μL/well) with six technical replicates. The fluorescence (excitation 488 nm, emission 525 nm) and optical density (OD_600_) of the samples were recorded every 1 h for 12 h using a microplate reader machine (Synergy H1MF, BioTek Instruments, Winooski, VT, USA).

### Pyocyanin measurement

Overnight cultures of *P. aeruginosa* grown in LB-MOPS broth were diluted into 4 mL *Pseudomonas* P broth (20 g/L pancreatic digest of gelatin, 1.4 g/L magnesium chloride, 10 g/L potassium sulfate) to reach a starting OD_600 _≈ 0.02. The bacteria were cultured at 37 °C for 24 h. The cells were then centrifuged at 12,000 g × 2 min. The culture supernatants were collected, and their OD values at 695 nm were photometrically determined. Pyocyanin production was estimated by determining OD_695_/OD_600_ values over time.

### Skim milk assay

Extracellular proteolytic activity of *P. aeruginosa* strains was evaluated using skim milk agar plates on which the bacteria form a protease-catalyzed clearing zone surrounding each colony. Individual colonies were grown on LB agar and then spotted on the skim milk agar plates (25% (v/v) LB, 4% (w/v) skim milk, 1.5% (w/v) agar). Colonies with extracellular proteolytic activity formed clearing zones after incubation at 37 °C for 24 h. The area of transparent zones reflecting extracellular proteolytic activity was quantified from captured photographs.

### Hydrogen cyanide production

Cells grown in LB-MOPS broth overnight were diluted to OD_600 _≈ 0.01 by fresh LB and continuously cultured to mid-log phase. Subsequently, cells were adjusted to OD_600 _≈ 0.02 and then 10 μL cells were transferred into 24-well plates containing 2% peptone agar. The plate was covered with a cyanide detection paper (Grade 3 MM Chr Cellulose Chromatography Paper, 3030–861, Whatman, UK) that was soaked in the HCN detection reagent: 10 mg copper (II) ethyl acetoacetate (41489, Alfa Aesar, Shanghai, China) and 10 mg 4,4’-methylenebis-(N, N-dimethylaniline) (A18466, Alfa Aesar, Shanghai, China) in 2 mL chloroform and cultured at 37 °C for 18–24 h. The production of hydrogen cyanide was quantified by measuring the intensity using ImageJ software (https://imagej.net/ij/index.html).

### Quantification of 3OC_12_-HSL

*P. aeruginosa* strains were cultured overnight in LB-MPOS broth and diluted to a starting OD_600_ ≈ 0.05. Cells were then grown in 4 mL LB at 37 °C for 18 h. AHLs were extracted with an equal amount of ethyl acetate. Relative quantification of 3OC_12_-HSL was performed using a reporter bioassay, in which 3OC_12_-HSL was detected by a ∆*lasI* strain containing P*lasB*-GFP.

### Mammalian cell cytotoxicity assay

CHO cells were cultured in RPMI medium (Thermo Fisher Scientific, Shanghai, China) supplemented with 10% FBS (Thermo Fisher Scientific, Shanghai, China), respectively at 5% CO2 and 37 °C. Exponentially growing *P*. *aeruginosa* bacteria cultured in casamino acids medium (OD_600 _≈ 0.5) were diluted with cell culture medium (OD_600 _≈ 0.1) and added to near-confluent CHO cells at a starting multiplicity of infection (MOI) ratio of 5:1. After incubation at 37 °C for 6 h, the extent of cell killing was determined by quantification of the release of lactate dehydrogenase into the cell culture supernatant using the LDH Cytotoxicity Detection Kit (Beyotime, Nantong, China). A549 cells are operated in the same way as described above.

### Monoculture experiments

Individual colonies of assayed strains were inoculated into a 3 mL LB-MOPS broth and grown overnight. Overnight cultures were then diluted to initial OD_600 _≈ 0.5, and one hundred microliters of the bacterial suspension was transferred into 3 mL of casein broth in 14 mL Falcon tubes (Corning). The cultures were incubated at 37 °C for 24 h, after which the tubes were photographed.

### Competition experiments

Assayed strains (one strain tagged with a gentamycin antibiotic resistance) were grown in casein broth. Overnight cultures of individual strains in MOPS-buffered LB were used as inocula for experiments and diluted to starting OD_600 _≈ 0.5 and dilute with 1% casein broth (OD_600 _≈ 0.02). In the case of co-culture experiments, the total starting OD_600_ is 0.02 and co-cultured at a ratio of 99:1. All fitness experiments are allowed to shake at 37 °C for 24 h. CFUs per mL were determined by dilution plating at t 0 and 24 h. Fitness was calculated according to the Malthusian growth model. Absolute fitness is expressed as the average rate of increase or Malthusian parameter (w), assuming that the number of competitors is X1 and the number of collaborators is Y1 after 24 h of cultivation, and the number of competitors and the number of collaborators are X0 and Y0 after 0 h, the calculation formula is as follows: w = ln(X1/X0)/ln(Y1/Y0). Relative fitness is expressed as the ratio of the Malthusian parameters (w) of two competing strains.

### Protein structural modeling

*P. aeruginosa* LasR structure was determined using the AlphaFold Protein Structure Database (ID: P25084) (https://alphafold.ebi.ac.uk/entry/P25084). The mutated residues of RpoA were visualized by the PyMOL molecular graphics system (version 2.5.2).

### Multiple sequence alignment

LasR protein homologues in different bacterial species used for sequence alignment are listed in S2 Table. Software Clustal Omega (https://www.ebi.ac.uk/Tools/msa/clustalo/) was applied for the sequence alignment.

### Statistical analysis

Statistical analyses were performed using Excel, GraphPad Prism 5 and R software.

## Supporting information

S1 Fig Localization of identified *lasR* mutations. Identified *lasR* mutations of *P. aeruginosa* isolates, evolved in casein broth, were mapped to the domains of the LasR protein (accession number: NP_252928.1). Top, nucleotide changes; bottom, amino acid changes.
(TIF)

S2 Fig Structural visualization of LasR variants. High-magnification images of substitutions in LasR are shown using PyMOL software.
(TIF)

S3 Fig Multiple sequence alignment of LasR homologs across different bacterial species. Sequence alignment was conducted using Clustal Omega. Amino acids are highlighted according to their conservation degrees. The amino acid substitutions of assayed LasR variants are highlighted in red. LBD, ligand binding domain; DBD, DNA binding domain.
(TIF)

S4 Fig Growth curve of assayed strains. Strains were grown in casamino acids medium. The OD_600_ was measured by using a microplate reader. The experiment was carried out in eight replicates and the log transformation of mean values is shown.
(TIF)

S5 Fig Growth of assayed strains in monoculture experiment. Culture tubes of individual assayed strains inoculated in casein broth, photographed after 24 hours of incubation.
(TIF)

S6 Fig Detection of QS activity in indicated strains. (A-C) Las- (A), Rhl- (B) and PQS-responsive (C) QS activities of the shown strains. Las-, Rhl- and PQS-responsive QS activities are reflected by the fluorescence levels of the expressed reporters P*lasB*-GFP, P*rhlA-*GFP and P*pqsA*-GFP, respectively. Fluorescence values, expressed as relative fluorescence units (GFP/OD_600_), were obtained from bacteria cultured for 18 h. Fluorescence values were obtained from bacteria cultured in casein broth for 18 h.
(TIF)

S7 Fig The *lasR* transcriptional expression in variant strains. The transcriptional level of *lasR* is estimated by the fluorescence signal of the P*lasR*-GFP. Fluorescence values, expressed as relative fluorescence units (GFP/OD_600_), were obtained from bacteria cultured for 18 h.
(TIF)

S8 Fig 3OC_12_-HSL promotes the LasR-responsive activity of LasR196 variant strains. LasR-responsive activity is indicated by the fluorescence levels of the expressed reporter P*lasB-*GFP. Fluorescence values, expressed as relative fluorescence units (GFP/OD_600_), were obtained from bacteria cultured with or without supplementation of 3OC_12_-HSL for 18 h. + C_12_, supplementation of 3OC_12_-HSL. Data are presented as means ± SD (*n *= 3).
(TIF)

S9 Fig The relative growth fitness of indicated variant strains. The relative growth fitness of indicated strains was calculated as the ratio of Malthusian growth parameters (*w*). Bacterial were co-cultured with the designated strain at a start ratio of 1:99 and grown in casein broth for 24 h. **, *P* < 0.01 by *t*-test.
(TIF)

S1 Table Identification of LasR variants during *P. aeruginosa* PAO1 in vitro evolution. (XLSX)

S2 Table LasR homolog sequences used in the study. (XLSX)

S3 Table Identification of LasR variants in *P. aeruginosa* strains obtained from native environments. (XLSX)

S4 Table Oligonucleotides used in this study. (XLSX)

S5 Table Strains and plasmids used in this study. (XLSX)

S6 Table Statistical analysis data. (XLSX)

S7 Table Source data. (XLSX)
