# Supplementary figures and images for: An anticipatory mechanism enhances the cooperative behaviors of quorum sensing mutants in Pseudomonas aeruginosa
Source: PLoS Pathog. 2025 Apr 15;21(4):e1013046. doi: 10.1371/journal.ppat.1013046 (PMC12021273; doi:10.1371/journal.ppat.1013046)

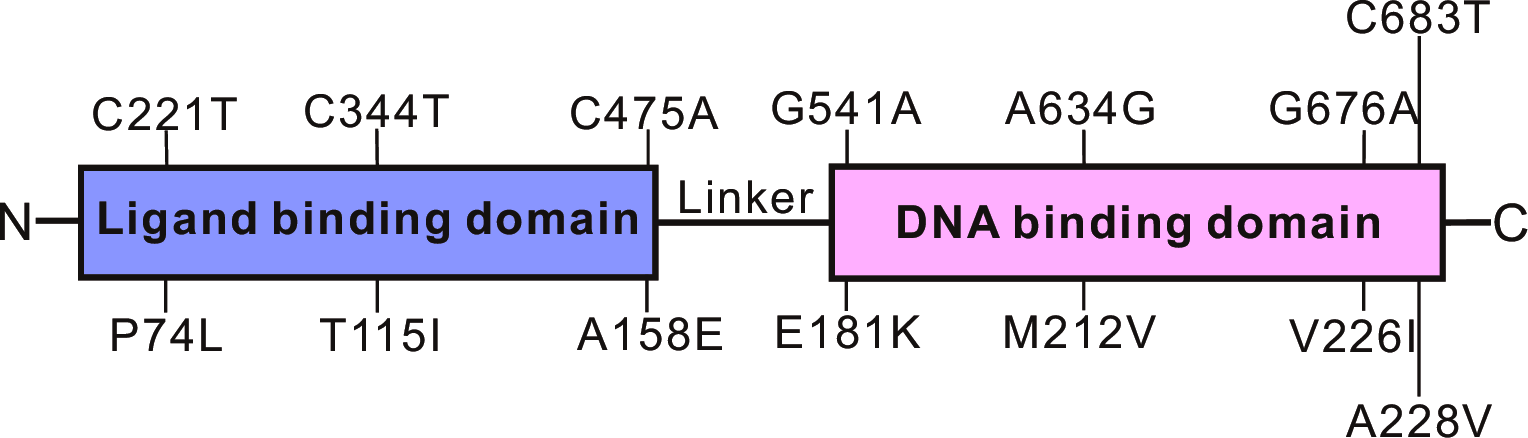

Supplement: S1 Fig — Identified lasR mutations of P. aeruginosa isolates, evolved in casein broth, were mapped to the domains of the LasR protein (accession number: NP_252928.1). Top, nucleotide changes; bottom, amino acid changes. (TIF) [file ppat.1013046.s001.tif]

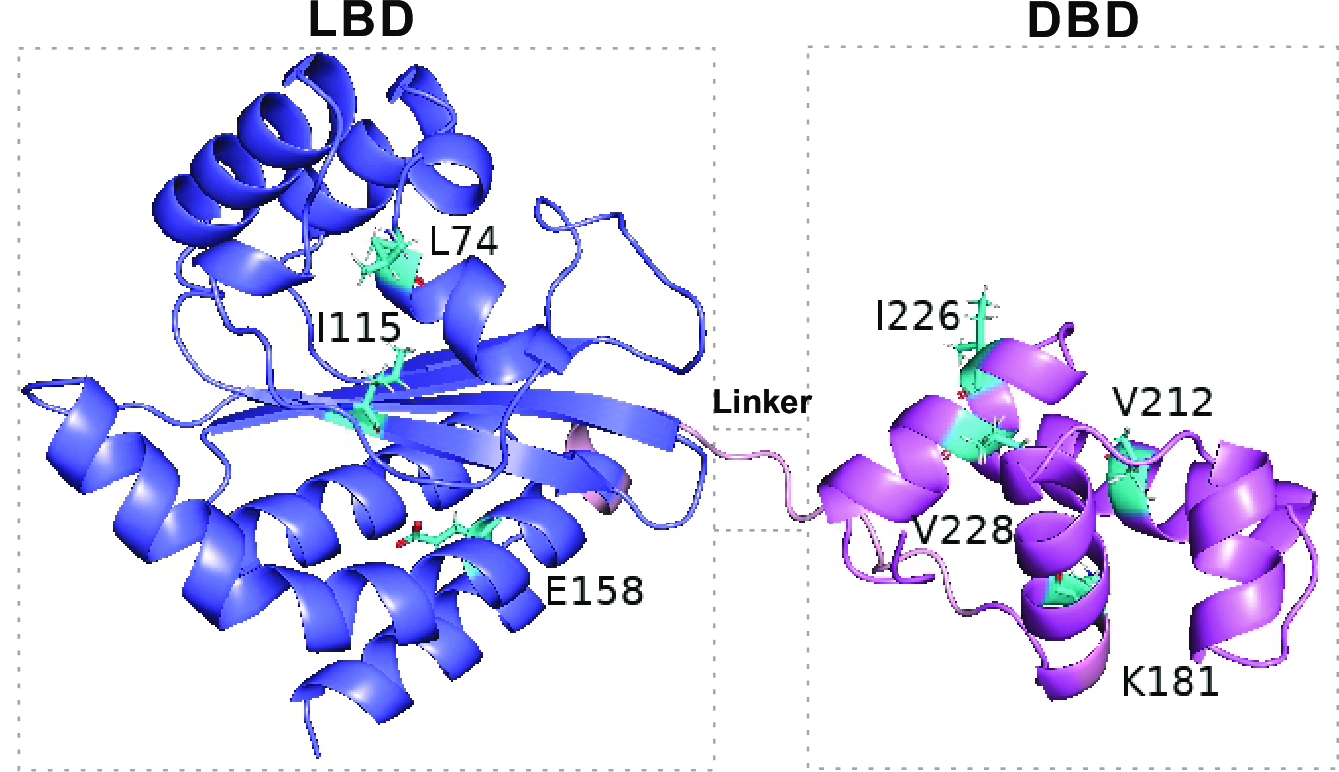

Supplement: S2 Fig — High-magnification images of substitutions in LasR are shown using PyMOL software. (TIF) [file ppat.1013046.s002.tif]

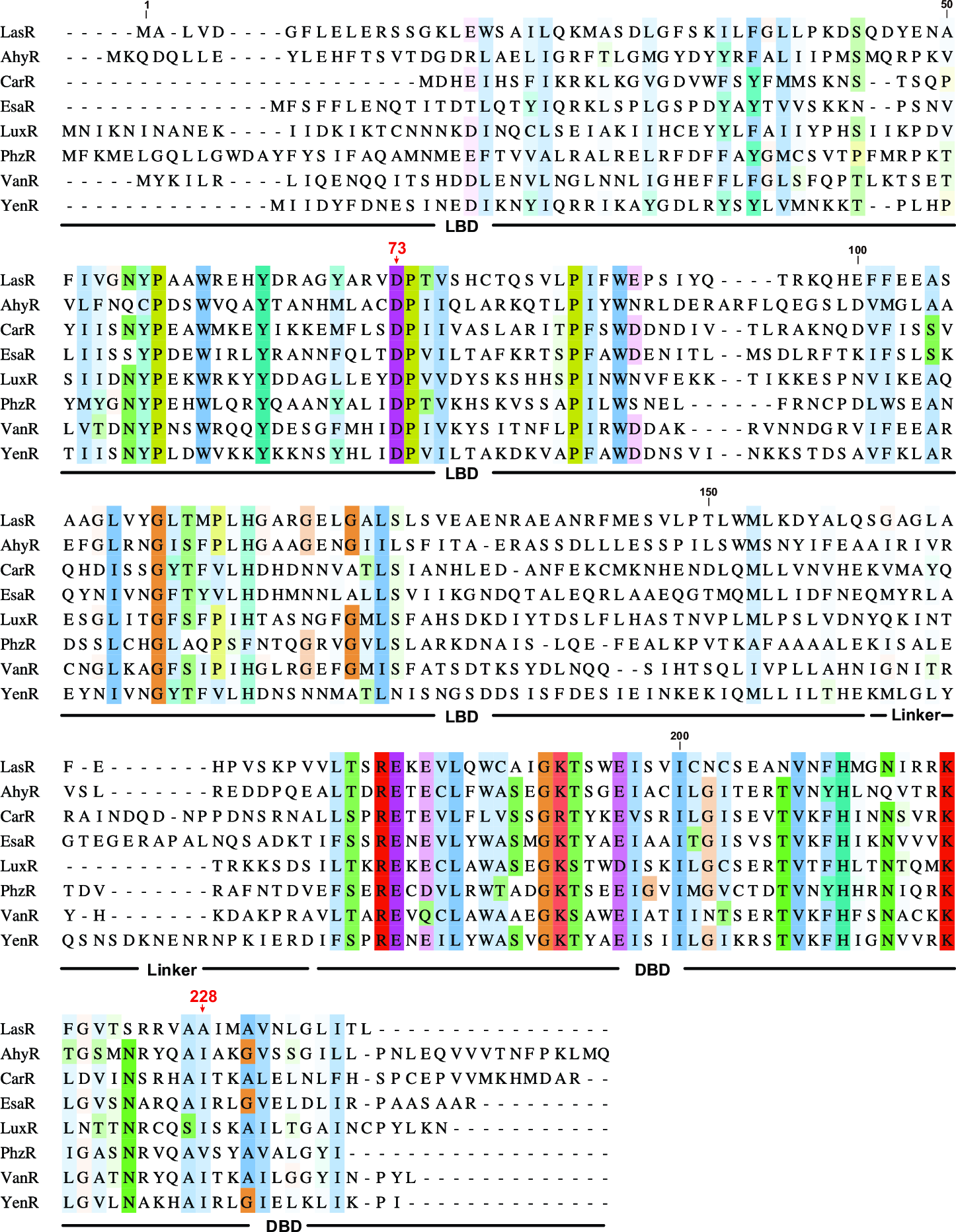

Supplement: S3 Fig — Sequence alignment was conducted using Clustal Omega. Amino acids are highlighted according to their conservation degrees. The amino acid substitutions of assayed LasR variants are highlighted in red. LBD, ligand binding domain; DBD, DNA binding domain. (TIF) [file ppat.1013046.s003.tif]

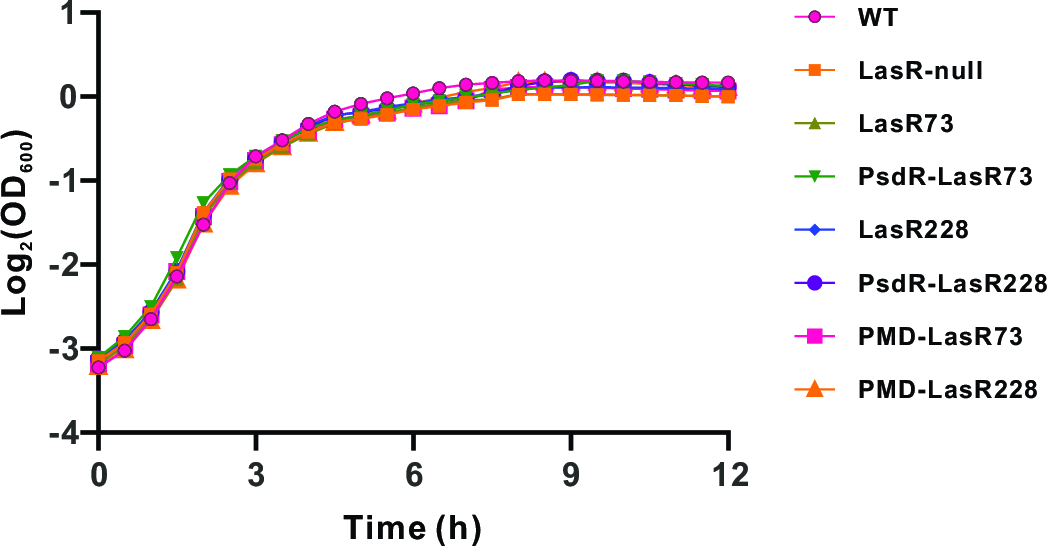

Supplement: S4 Fig — Strains were grown in casamino acids medium. The OD600 was measured by using a microplate reader. The experiment was carried out in eight replicates and the log transformation of mean values is shown. (TIF) [file ppat.1013046.s004.tif]

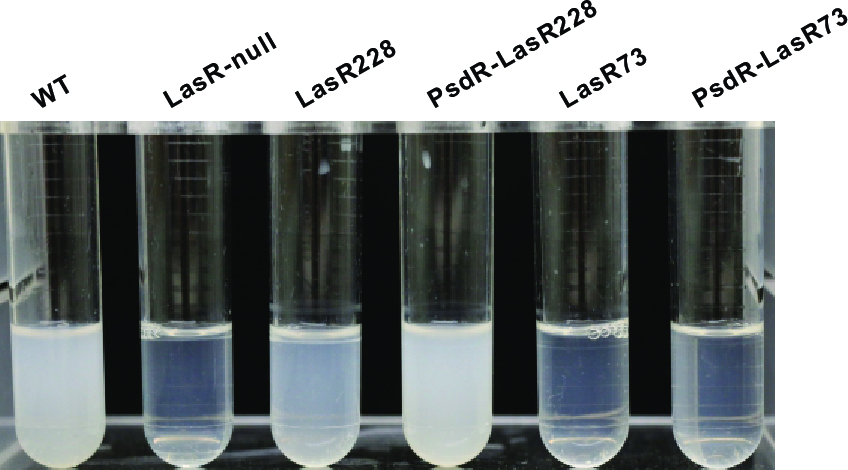

Supplement: S5 Fig — Culture tubes of individual assayed strains inoculated in casein broth, photographed after 24 hours of incubation. (TIF) [file ppat.1013046.s005.tif]

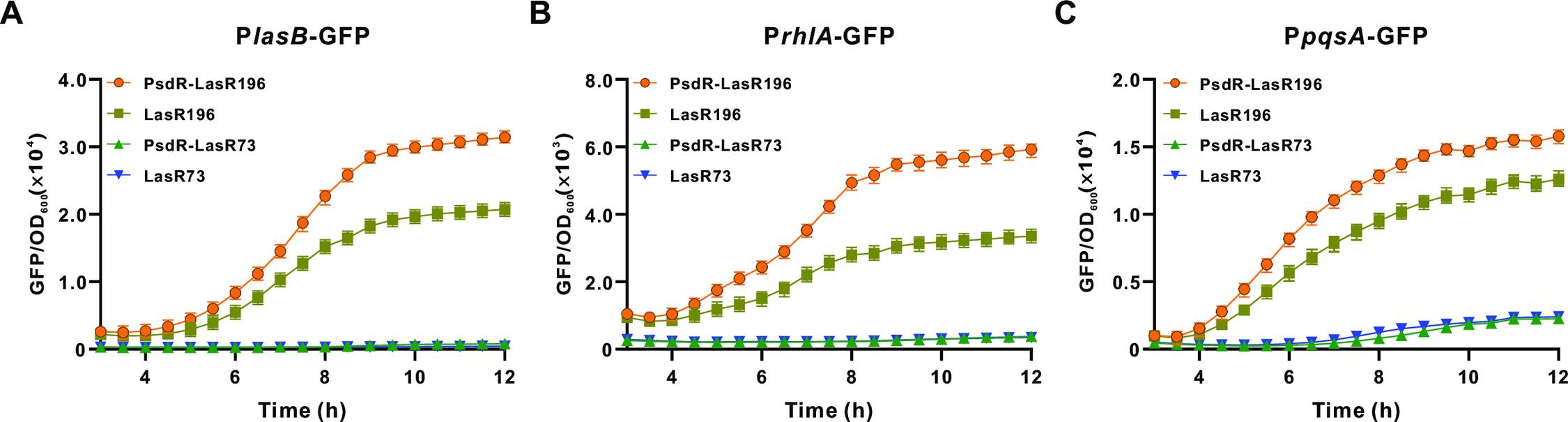

Supplement: S6 Fig — (A-C) Las- (A), Rhl- (B) and PQS-responsive (C) QS activities of the shown strains. Las-, Rhl- and PQS-responsive QS activities are reflected by the fluorescence levels of the expressed reporters PlasB-GFP, PrhlA-GFP and PpqsA-GFP, respectively. Fluorescence values, expressed as relative fluorescence units (GFP/OD600), were obtained from bacteria cultured for 18 h. Fluorescence values were obtained from bacteria cultured in casein broth for 18 h. (TIF) [file ppat.1013046.s006.tif]

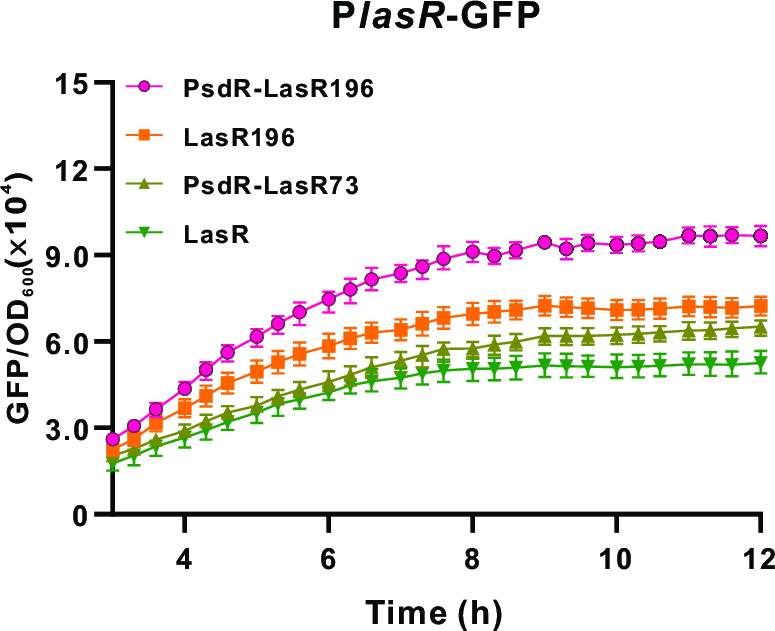

Supplement: S7 Fig — The transcriptional level of lasR is estimated by the fluorescence signal of the PlasR-GFP. Fluorescence values, expressed as relative fluorescence units (GFP/OD600), were obtained from bacteria cultured for 18 h. (TIF) [file ppat.1013046.s007.tif]

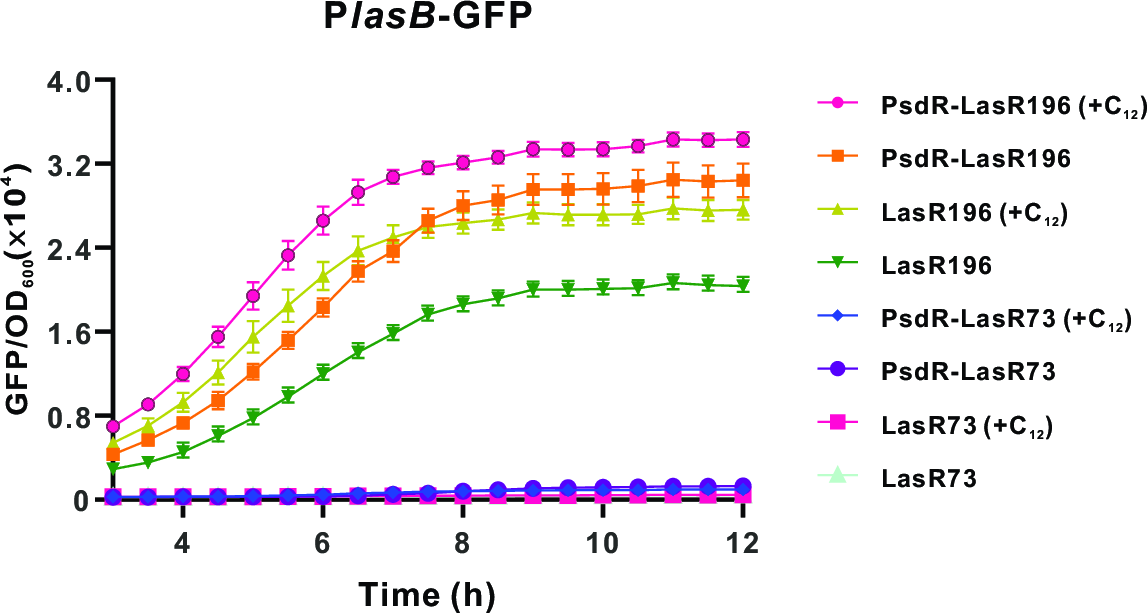

Supplement: S8 Fig — LasR-responsive activity is indicated by the fluorescence levels of the expressed reporter PlasB-GFP. Fluorescence values, expressed as relative fluorescence units (GFP/OD600), were obtained from bacteria cultured with or without supplementation of 3OC12-HSL for 18 h. + C12, supplementation of 3OC12-HSL. Data are presented as means ± SD (n = 3). (TIF) [file ppat.1013046.s008.tif]

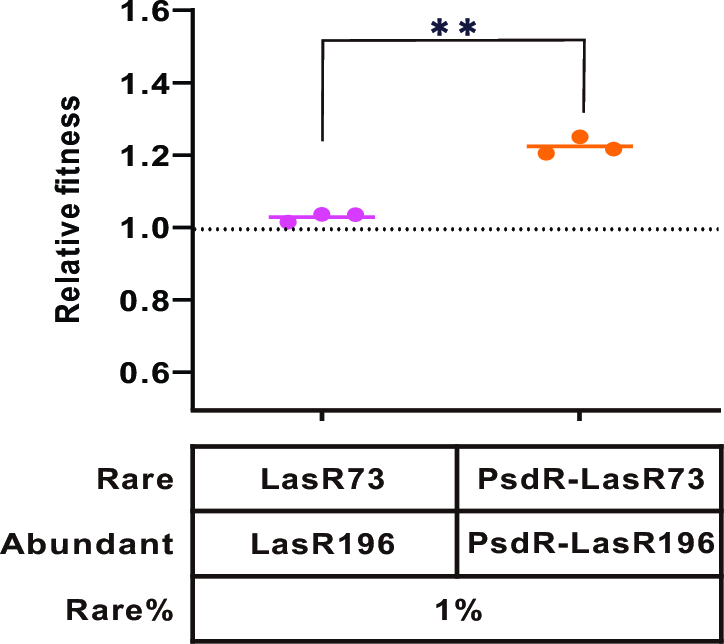

Supplement: S9 Fig — The relative growth fitness of indicated strains was calculated as the ratio of Malthusian growth parameters (w). Bacterial were co-cultured with the designated strain at a start ratio of 1:99 and grown in casein broth for 24 h. **, P < 0.01 by t-test. (TIF) [file ppat.1013046.s009.tif]
